# Supplementary material for: Inferring the genetic basis of sleep states in Drosophila melanogaster using hidden Markov models
Source: bioRxiv. 2026 Jan 14:2026.01.14.699526. Preprint. [Version 1] doi: 10.64898/2026.01.14.699526 (PMC12919566; doi:10.64898/2026.01.14.699526)
Supplement: Supplement 2 [file NIHPP2026.01.14.699526v1-supplement-2.pdf]

bioRxiv preprint doi: https://doi.org/10.64898/2026.01.14.699526; this version posted January 14, 2026. The copyright holder for this preprint (which was not certified by peer review) is the author/funder. This article is a US Government work. It is not subject to copyright under 17 USC 105 and is also made available for use under a CC0 license.

SUPPLEMENTARY FIGURES

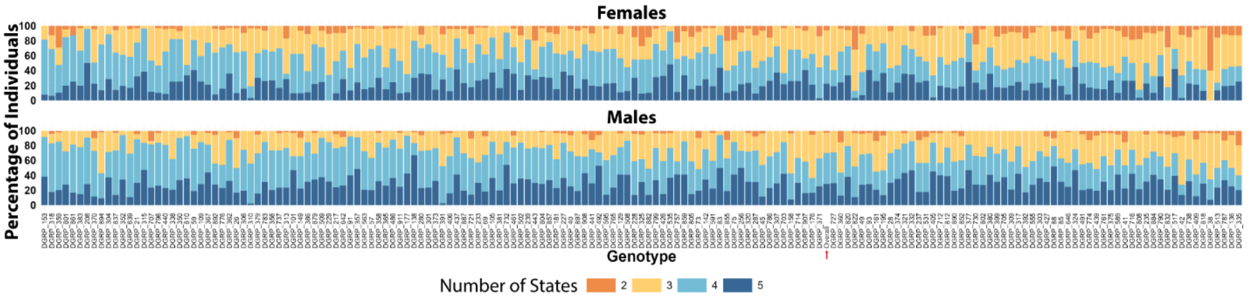

Supplementary Figure 1. Percentage of individuals fitting 2, 3, 4, and 5 sleep/wake states across the DGRP. Among all 168 genotypes in the DGRP, 4.4% showed best fit to 2 states, 30.5% to 3 states, 39.8% to 4 states, and 25.3% to 5 states. Numbers of individuals indicating best fit to each number of states are represented as percentages to compare across sexes (top row females and bottom row males) and genotypes. All 168 genotypes are plotted across the *x*-axis. The "overall" on the x-axis marked with a red arrow represents the mean of all 168 genotypes.

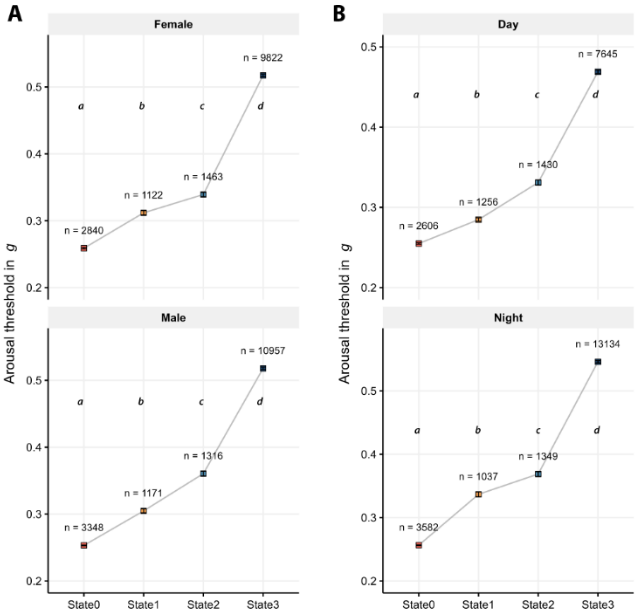

Supplementary Figure 2. Arousal threshold probing of 28 DGRP lines detects four distinct sleep and waking states irrespective of sex and time of day. A and **B** are mean arousal threshold values for flies predicted to be in each sleep/wake state using the HMM in different sexes, and different times of day, respectively. The error bars are ± SE. Mean values were significantly different from one another as indicated by letters ($P < 0.05$ and post-hoc Dunn's analysis).

bioRxiv preprint doi: https://doi.org/10.64898/2026.01.14.699526; this version posted January 14, 2026. The copyright holder for this preprint (which was not certified by peer review) is the author/funder. This article is a US Government work. It is not subject to copyright under 17 USC 105 and is also made available for use under a CC0 license.

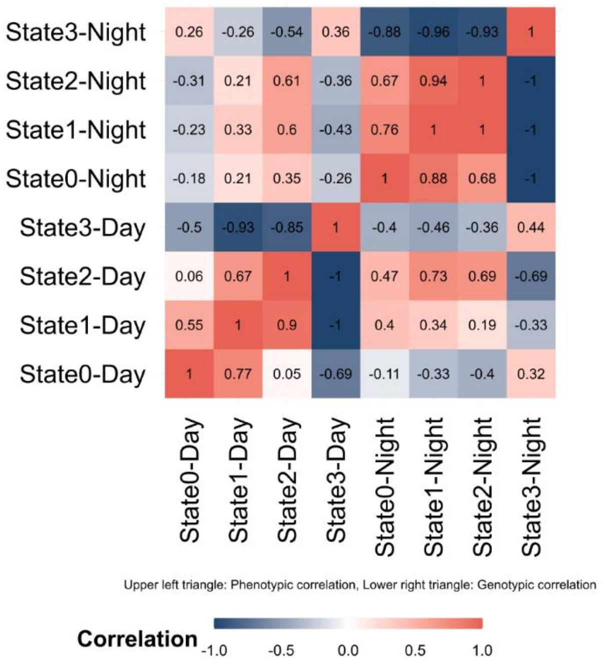

Supplementary Figure 3. Heatmap of phenotypic and genotypic correlation coefficients among time spent in 4 sleep/wake states in day and night. The phenotypic (upper left triangle) and genotypic (lower right triangle) correlation coefficients were calculated as described in the *Materials and Methods* section. Correlation values range from +1 (red) to -1 (blue). In general, for a given state, the correlation between time spent in day and night, while statistically significant in most cases, were lower than correlations within a given phase, indicating that the genetic architecture between time spent in night states and day states is likely to be different.

bioRxiv preprint doi: https://doi.org/10.64898/2026.01.14.699526; this version posted January 14, 2026. The copyright holder for this preprint (which was not certified by peer review) is the author/funder. This article is a US Government work. It is not subject to copyright under 17 USC 105 and is also made available for use under a CC0 license.

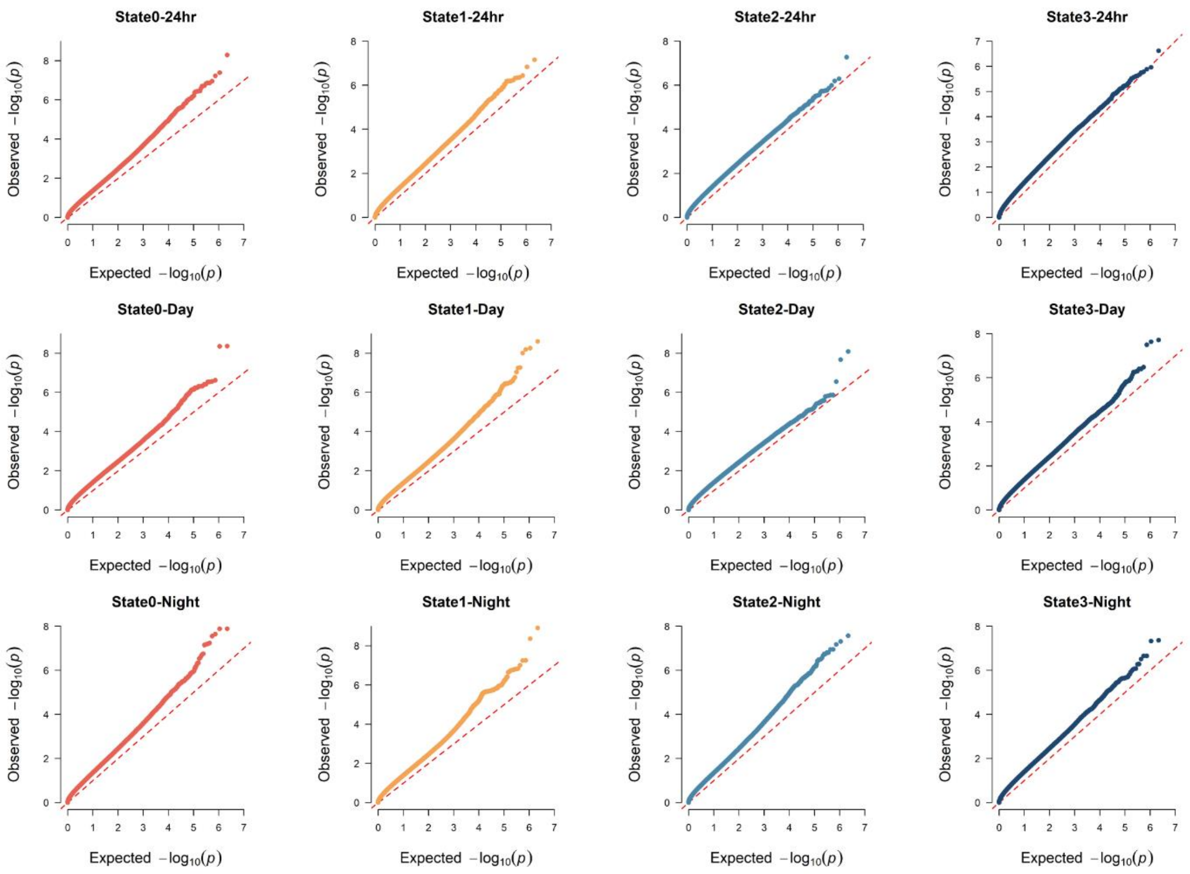

Supplementary Figure 4. Quantile-Quantile (Q-Q) plots illustrating observed vs expected distributions of negative log-transformed *P*-values (*-log_{10}(P)*) from the GWASs for each trait. The top, middle, and bottom rows show Q-Q plots for into 24-hour, daytime, and nighttime states, respectively. Each point represents a SNP tested in the analysis. The dashed red lines indicate the expected distribution under the null hypothesis, highlighting deviations indicative of significant genetic associations.

bioRxiv preprint doi: https://doi.org/10.64898/2026.01.14.699526; this version posted January 14, 2026. The copyright holder for this preprint (which was not certified by peer review) is the author/funder. This article is a US Government work. It is not subject to copyright under 17 USC 105 and is also made available for use under a CC0 license.

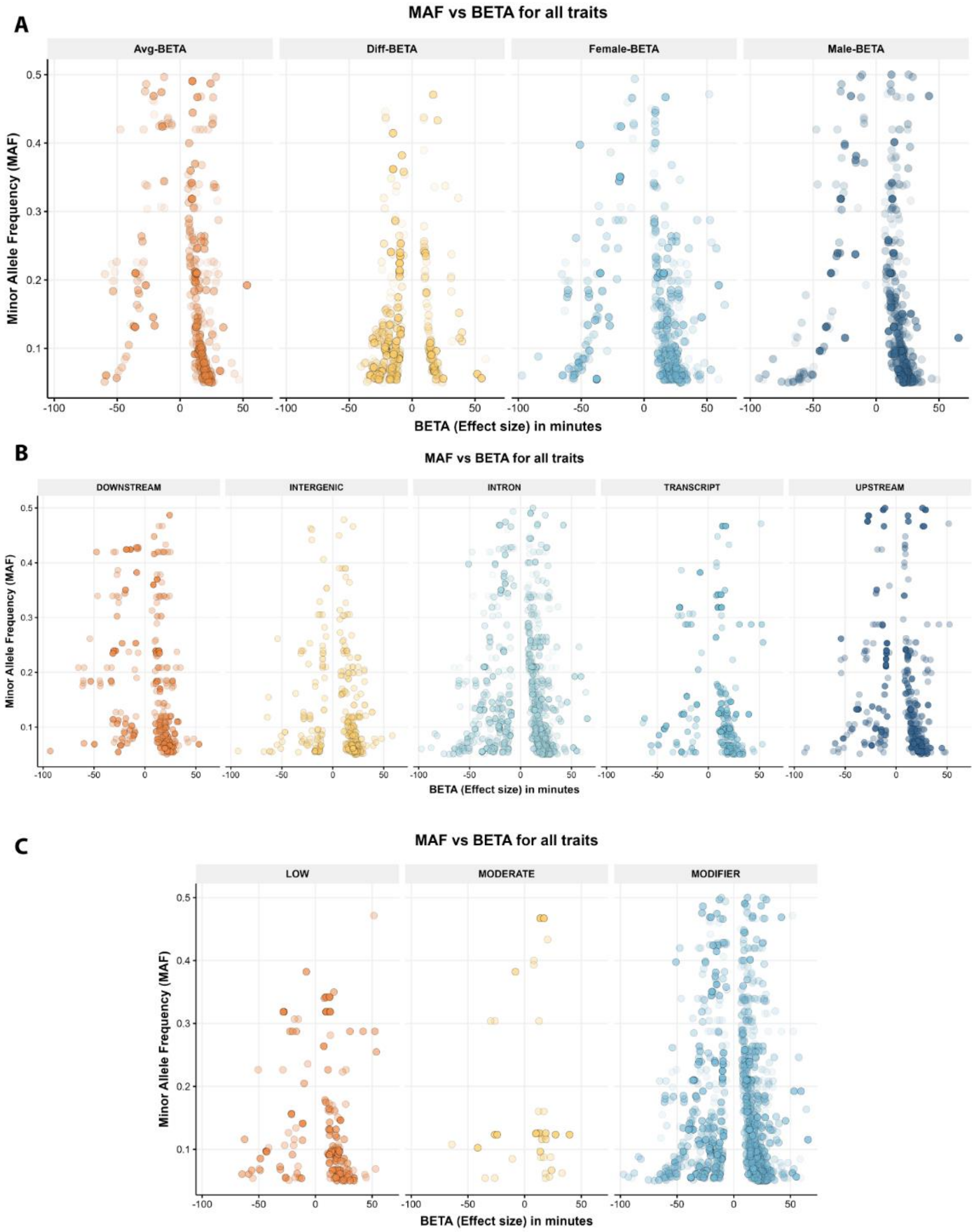

Supplementary Figure 5: Relationship between Minor Allele Frequency (MAF) and effect sizes (BETA, measured in minutes) for polymorphisms associated with different traits. (A) Comparison of MAF against effect sizes for average (Avg-BETA), differential (Diff-BETA), female-specific (Female-BETA), and male-specific (Male-BETA) polymorphisms. **(B)** Effect size distribution categorized by genomic annotations: downstream, intergenic, intron, transcript, and upstream regions. **(C)** Distribution of effect sizes grouped by predicted functional impact: low, moderate, and modifier categories. Each data point represents an individual polymorphism, highlighting the variation in allele frequencies and their phenotypic effects across traits and genomic contexts.

bioRxiv preprint doi: https://doi.org/10.64898/2026.01.14.699526; this version posted January 14, 2026. The copyright holder for this preprint (which was not certified by peer review) is the author/funder. This article is a US Government work. It is not subject to copyright under 17 USC 105 and is also made available for use under a CC0 license.

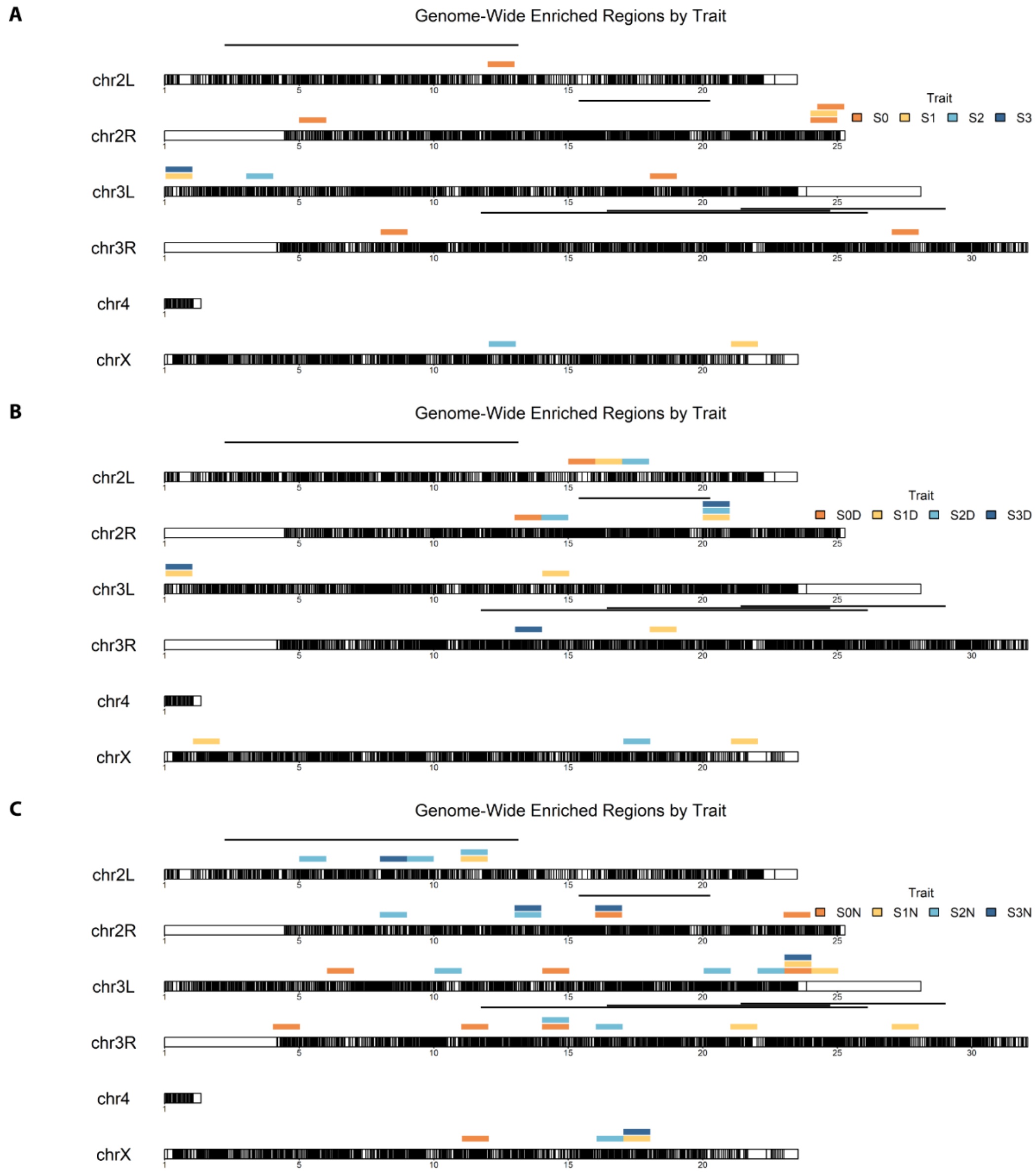

Supplementary Figure 6: Genome-wide representation of significant SNP enriched regions across chromosomes in the DGRP. Panels depict genomic regions significantly enriched in SNPs associated with time spent in 4 sleep/wake states **(A)** in the 24-hour period, **(B)** in the day, and **(C)** in the night. Colored bars indicate a 1-Mb stretch of significant genomic regions enriched for each trait (S0: State0, S1: State1, S2: State2, and S3: State3), mapped onto chromosomal arms (*2L*, *2R*, *3L*, *3R*, *4*, and *X*). The black bars show the positions of 5 major chromosomal inversions present in the DGRP.
